# Lack of functional brain connectivity was associated with poor inhibition in children with attention-deficit/hyperactivity disorder using near-infrared spectroscopy

**DOI:** 10.3389/fpsyt.2023.1221242

**Published:** 2023-07-12

**Authors:** Wenjing Liao, Longfei Cao, Lingli Leng, Shaohua Wang, Xinyu He, Yusang Dong, Rongwang Yang, Guannan Bai

**Affiliations:** ^1^Department of Psychology, Children’s Hospital, Zhejiang University School of Medicine, National Clinical Research Center for Child Health, Hangzhou, China; ^2^Centre for Cognition and Brain Disorders, Affiliated Hospital of Hangzhou Normal University, Hangzhou, China; ^3^Department of Sociology, Zhejiang University, Hangzhou, China; ^4^Affiliated Mental Health Center, Zhejiang University School of Medicine (Hangzhou Seventh People’s Hospital), Hangzhou, China; ^5^Department of Child Health Care, Children’s Hospital, Zhejiang University School of Medicine, National Clinical Research Center for Child Health, Hangzhou, China

**Keywords:** attention deficit hyperactivity disorder, brain, functional connection, inhibition function, near-infrared spectroscopy, children

## Abstract

**Objectives:**

The present study aimed to evaluate the characteristics of functional brain connectivity in the resting state in children with attention deficit hyperactivity disorder (ADHD) and to assess the association between the connectivity and inhibition function using near-infrared spectroscopy (NIRS).

**Methods:**

In total, 34 children aged 6–13 diagnosed with ADHD were recruited from Hangzhou Seventh People’s Hospital. In comparison, 37 healthy children were recruited from a local primary school as controls matched by age and sex. We used NIRS to collect information on brain images. The Stroop test assessed inhibition function. We compared the differences in functional brain connectivity in two groups by analyzing the resting-state brain network. Pearson partial correlation analysis was applied to evaluate the correlation between functional brain connectivity and inhibition in all the children.

**Results:**

Compared with the control group, results of NIRS images analysis showed that children with ADHD had significantly low functional brain connectivity in regions of the orbitofrontal cortex, left dorsolateral prefrontal cortex, left pre-motor and supplementary motor cortex, inferior prefrontal gyrus, and right middle temporal gyrus (*p* = 0.006). Inhibition function of children with ADHD was negatively correlated with functional brain connectivity (*p* = 0.009), while such correlation was not found in the control group.

**Conclusion:**

The present study demonstrated that children with ADHD had relatively low connectivity in several brain regions measured at the resting state. Our results supported the evidence that lack of functional brain connectivity was associated with impaired inhibition function in children with ADHD.

## 1. Introduction

Attention-deficit/hyperactivity disorder (ADHD) is among childhood’s most common mental health disorders. A recent meta-analysis of more than 100 studies estimated that the global prevalence rates of ADHD are around 3.4–5.3% (1). In China, a national survey of psychiatric diseases reported that the prevalence of ADHD in school-aged children was 10.2%, while boys having a relatively higher rate (2). The typical symptoms are inattention, hyperactivity, and impulsivity, which are inappropriate for the age, and often lead to impairment in their academic performance, work productivity, and social skills in later life (3, 4). Empirically, the phenotypes of ADHD are variant in the population and may be related to genetic and neurological factors during brain development, including the exposure to toxins, and deprived social-economic resources in the early period of life (4). ADHD is a consequence of various executive function deficits; in particular, it is primarily characterized by a deficiency in behavioral inhibition that is the ability to inhibit irrelevant or interfering information and impulses which results in further impairments in other executive functions, such as working memory and self-regulation (3).

Despite abnormal behaviors, structural and functional changes of the brain are commonly observed among individuals with ADHD, such as a decrease in the volume of the white matter, smaller gray matter volume, focal thinning in bilateral frontal regions, and the right cingulate cortex, as well as the decreased functional connectivity (5, 6). In recent years, the rapid development of brain imaging techniques (e.g., magnetic resonance imaging, MRI) allows closer observation of the ADHD brain. For instance, recent studies have shown that ADHD is characterized by multiple structural and functional abnormalities in the neural network, including alterations in fronto-parieto-temporal, fronto-cerebellar, and even front-limbic networks (5–7). MRI, especially functional MRI (fMRI), has been widely used in basic medical and clinical research as well as clinical practice to investigate the structure and function of brain. However, it is a great challenge for children, especially the very young ones, who have to stay in a closed and dark space for a considerable period of time during the scanning. They are required to remain still because if they move, the imaging is not precise. Additionally, the costs of MRI, functional MRI, and rest-stating MRI are high. In response to these methodological limitation, functional near-infrared spectroscopy (fNIRS), was introduced into the scientific community two decades ago. It is an optic-based tool for measuring neural function. It has the advantages of being less susceptible to head movement artifacts, and having a non-intrusive acquisition environment and good portability (8). fNIRS has been frequently used to explore the neural basis of cognition associated with ADHD, such as executive function, facial expression recognition, and emotion regulation (8–11).

Resting-state functional near-infrared spectroscopy (rs-fNIRS) imaging is a natural imaging paradigm with many advantages over task-state fNIRS (12–14). The rs-fNIRS is simple to operate and easy to handle in clinical practice, especially for pediatric patients who are difficult to keep stable and tend to move. The rs-fNIRS technique can reveal changes in the brain network regarding normal development and psychopathological status (12, 13, 15, 16). A study by Wang et al. (7) demonstrates the feasibility and potential of applying fNIRS to ADHD.

In the present study, we applied the rs-NIRS among children with ADHD to (1) investigate the altered functional brain connectivity by comparing the children with ADHD and healthy controls and (2) assess the association between functional brain connectivity and inhibition in children with ADHD.

## 2. Materials and methods

### 2.1. Study participants

This study was a cross-sectional study by enrolling children with ADHD who visited the Hangzhou Seventh People’s Hospital outpatient clinic from July 2019 to July 2020. The inclusion criteria were children (1) with a diagnosis of ADHD made by a psychiatrist according to the Diagnostic and Statistical Manual of Mental Disorders-Fourth Edition-Text Revision (DSM-IV) using a semi-structured interview of the Clinical Diagnostic Interview Scale (17, 18); (2) with an IQ ≥ 80 according to the evaluation by the Chinese-Wechsler Intelligence Scale for Children (C-WISC) (19); (3) aged between 6 and 13 years old; (4) right-handed; and (5) who did not take any central stimulants, atomoxetine or other psychiatric drugs. The reason why we chose the age range as 6–13 years was based on findings from the Chinese Color Nest Project (CCNP) that aimed to describe normative charts for brain structure and function across the human lifespan, and link age-related changes in brain imaging measures to psychological assessments with a longitudinal study design (20). Based on the ongoing CCNP, Dong et al. (21) found that wide connectome-level gradients gradually matured throughout development, reaching a peak around the age of 13 years. The exclusion criteria were children (1) with mental disorders consistent with DSM-IV Axis I diagnosis; (2) with severe physical illnesses such as neurological disorders, history of severe traumatic brain injury (causing coma), color blindness, or hearing deficits that significantly affect auditory comprehension; and (3) who were not able to understand and use Mandarin.

The control group were students selected from a local primary school between July 2019 to July 2020 according to the Clinical Diagnostic Interview Scale (CDIS) for children (17, 18). A psychiatrist conducted the screening and excluded cases with mental health and neurological concerns. The inclusion criteria were children (1) with IQ ≥ 80 according to the evaluation by the Chinese-Wechsler Intelligence Scale for Children (C-WISC) (19); (2) whose ages between 6 and 13 years old and whose sex ratio matched the study group. Children were excluded if (1) they reported more than four items of attention deficit and/or hyperactive impulsivity on the ADHD-IV symptom scale; (2) they currently or previously had psychiatric diseases, significant physical and neurological diseases, or disorders. All the selected participants in the control group were right-handed, with normal naked-eye vision or corrected vision, and they had no color weakness, glaucoma, or other abnormalities.

The study was approved by the medical ethics committee of the Hangzhou Seventh People’s Hospital, and the guardians of all the participants in the study signed written consent.

### 2.2. Measurement

#### 2.2.1. Children’s clinical diagnostic interview scale (CDIS)

This scale is a semi-deterministic diagnostic interview scale developed by the Childhood Disorders Group in the United States. It was translated in Chinese and revised by Li Yang and Yufeng Wang with good reliability and validity (18). The sensitivity of CDIS was 97.2%, and the specificity was 100%. The test-retest reliability was 0.89, and the inter-rater agreement Kappa was 0.74.

#### 2.2.2. Chinese revised wechsler intelligence scale for children (C-WISC)

This scale assesses the total intelligence quotient (IQ), verbal IQ, and operational IQ of children aged 6–16. The reliability of each sub-test score is high (around 0.80), and the reliability of the IQ score is above 0.90 (19). The test-retest reliability scores were between 0.59 and 0.86. The construct validity is good. A higher score indicates a higher IQ.

#### 2.2.3. Assessment of the inhibition

We used the classical version of the Stroop Color-Word Test to assess the inhibition function (22, 23). The test consists of four parts and three cards (21 × 29.7 cm) (24). The subject was asked to name 30 stimuli in a 10 × 3 matrix as quickly and correctly as possible (24, 25). Test 1 requires the subject to read Chinese words that mean red, green, yellow, and blue printed in black ink in a random order. Test 2 requires the subject to name the color of blocks printed in red, green, yellow, and blue. Test 3 involved a color-word card, and the subject was required to name the words of the color-content that did not match the color words. In Test 4, the same color-word card was used, but the subject was required to name the colors. The time to complete each test (in seconds) and the number of errors were recorded. The time taken to complete Test 3 was subtracted from that for Test 1 to indicate color interference, and the time taken to complete Test 4 was subtracted from that for Test 2 to indicate word interference (24). A shorter time to complete the task and fewer errors indicate a better function of inhibition. Supplementary Figure 1 shows an example of cards and explains the application procedure of the Stroop Color-Word Test.

#### 2.2.4. Information on imaging

We used the 33-guide Near-Infrared Spectroscopy (NIRS) system (ETG-4000 NIRS instrument, Hitachi, Japan) to determine the hemodynamic changes in the prefrontal and temporal lobes of the brain during the resting state of the subjects. Subjects’ performance and changes in signal recordings during the experiment were observed in real time by the researcher, and artificial interference signals were flagged. Data or segments with significant artificial interference were excluded during fNIRS data preprocessing. fNIRS signals were acquired at a frequency of 10 Hz.

### 2.3. Statistical analysis

Analysis was performed using the IBM SPSS Statistics for Windows, Version 22.0 (Armonk, New York: IBM Corp). Firstly, we conducted a descriptive analysis. Means and standard deviations (SDs) were calculated for continuous variables. The categorical variables were expressed as numbers and percentages. Secondly, we applied an independent two sample t-tests and one-way Analysis for Variance (ANOVA) to compare the differences across groups; and we conducted the Chi-square (χ^2^) tests to compare the differences in the numbers and percentages between the two groups in terms of the categorical variables. Thirdly, because the data was not normally distributed, we used Tukey method to transfer data into rank and made it conform to the normal distribution. Then, we included the normal-distributed variable in the multivariate ANOVA model by using sex, age, and IQ as covariates.

The images were analyzed by the Network-Based Statistic (NBS), an appropriate approach to compare the differences in the functional connectivity networks between the study group and the control group. First, a functional connectivity matrix was generated for each subject, and then a two-sample *t*-test was done for each edge to develop a binary network. According to the threshold of *p*-value, the network components were then divided into half, and a permutation test was done for component size to derive a significant difference network. In the analysis of variance, we adjusted the regression model by age, sex, and IQ.

We conducted Pearson’s partial correlation analysis to assess the association between functional brain connectivity and ADHD inhibition in the two groups separately, adjusted by age, sex, and IQ. The statistical significance is indicated when the *p-*value is less than 0.05.

## 3. Results

### 3.1. General characteristics of the study population

In total, 34 children with ADHD were enrolled in the study group, and 37 children were included in the control group. The mean age of children with ADHD was 8.90 (SD:0.40) years; 33 (97%) were males; the mean IQ was 106.6 (SD: 2.5), and 28 (82.3%) children had inattentive subtype (ADHD-I), 2 (5.9%) children with hyperactive/impulsive subtype (ADHD-H), and 4 (11.8%) combined subtype (ADHD-C). The mean age of children in the control group was 8.60 (SD:0.40) years; 35 (94.6%) were males; the mean IQ was 115.9 (SD: 2.5). There were no statistically significant differences in age, sex, and IQ between the ADHD group and the control group (*p*-values > 0.05).

### 3.2. Comparison of results of the Stroop test between the ADHD group and control group

Table 1 presents the performance results of the Stroop test in two groups. Multivariate ANOVA analysis shows differences in the inhibition function between the two groups. Children with ADHD took longer to complete test 4 than those in the control group (*p* = 0.03).

**TABLE 1:** Comparison of results of Stroop test between the attention deficit hyperactivity disorder (ADHD) group and the control group.

Item	ADHD group	Control group	*F*	*p*
Time of test 1	15.5 (24.0)	19.0 (6.0)	0.07	0.79
Time of test 2	27.0 (15.0)	24.0 (12.0)	2.40	0.13
Time of test 3	20.0 (40.0)	24.0 (9.0)	0.04	0.85
Time of test 4	54.0 (36.0)	46.0 (28.0)	5.40	0.03
Time of (test 3-test1)	4.0 (12.8)	5.0 (7.0)	0.14	0.71
Time of (test 4-test 2)	21.0 (19.8)	21.0 (17.0)	0.15	0.70

### 3.3. Characteristics of brain connectivity at resting state in children with ADHD

**FIGURE 1:**
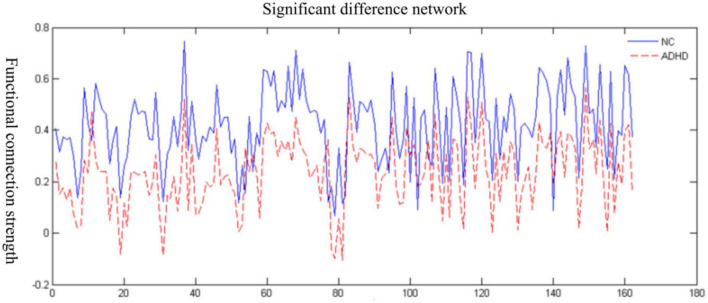
Changes in the functional brain connectivity strength in the attention deficit hyperactivity disorder (ADHD) group and the normal control (NC).

We used a network-based statistical (NBS) approach to analyze the brain connectivity function at the resting state in children with and without ADHD. The results are presented in Figure 1. The strength of brain connectivity was significantly weaker in the ADHD group compared to children in the control group (*p* = 0.007).

**FIGURE 2:**
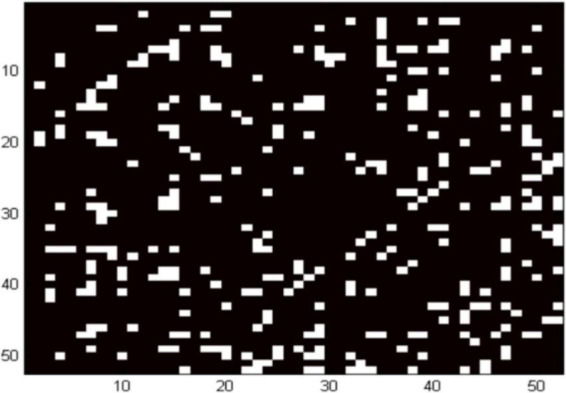
Location of networks at the resting date in children with ADHD. White areas indicate channels with decreased functional brain connectivity.

Figure 2 shows the locations of the channels with significantly weaker functional brain connectivity in the ADHD group compared to the control group. Combined with the cortical localization of each channel of fNIRS, it is clear that the functional connectivity between the orbitofrontal cortex, left dorsolateral prefrontal, left premotor and supplementary motor areas, inferior frontal gyrus, and right middle temporal gyrus is diminished in the ADHD group compared with those in the control group (*p* = 0.01).

### 3.4. Association between brain connectivity and inhibition

Table 2 shows the results of the correlation analysis in the ADHD group and the control group. Time of test 2 (*r* = −0.45, *p* = 0.006) and time of test 4 (*r* = −0.44, *p* = 0.009) were negatively correlated with the whole-brain resting-state functional connectivity (RSFC) in the Stroop test, respectively. In the control group, we did not find a statistically significant correlation between the inhibition test scores and the whole-brain RSFC.

**TABLE 2:** Association of Stroop test scores with whole-brain resting-state functional connectivity (RSFC) in ADHD group and the control group.

Stroop test	RSFC in the ADHD group		RSFC in the control group	
	**Pearson *r***	***p***	**Pearson *r***	***p***
Time of test 1	−0.19	0.27	−0.05	0.81
Time of test 2	−0.45	0.006	−0.06	0.80
Time of test 3	−0.24	0.17	−0.02	0.97
Time of test 4	−0.44	0.009	−0.07	0.71
Time of (test 3-test1)	0.02	0.90	0.03	0.89
Time of (test 4-test 2)	0.01	0.94	−0.11	0.36

## 4. Discussion

In the present study, we found that functional brain connectivity was diminished in specific brain regions in children within the ADHD group compared to the control group using the resting-state functional near-infrared spectroscopy (rs-fNIRS) and brain network statistics (NBS). The function of brain connectivity was weaker in children with ADHD than those in the control group. More specifically, the areas with weaker signals are mainly located in the prefrontal cortex, premotor areas, and supplementary motor areas, primarily related to the executive control networks and sensorimotor networks. Studies on imaging the ADHD brain have primarily focused on functional magnetic resonance imaging (MRI) techniques. A recent meta-analysis of 96 relevant studies demonstrated reduced activity in the left inferior frontal gyrus in children with ADHD at both task and resting states (26). We also observed that the functional connectivity of the inferior frontal gyrus was diminished bilaterally in the ADHD group, suggesting that the inferior frontal gyrus may be useful for diagnosis and target treatment. Another meta-analysis reviewed the findings of functional brain networks in ADHD at a resting state and yielded different results in children and adults (27). In children, networks related to executive inhibition (frontoparietal network) and attention (ventral attention network) were hyperactivated, and the default network, ventral attention, and sensorimotor networks were hyperactivated compared to controls. At the same time, in adults, ADHD-related hypoactivation was mainly observed in the frontoparietal network, while hyperactivation was in the visual, dorsal attention, and default networks (28, 29). We did not observe similar results, which may be related to the fact that the spatial resolution of NIRS is not as good as fMRI and that we had a relatively small sample.

We found a negative correlation between the inhibition and whole-brain RSFC in the ADHD group; that is, the poorer the inhibition function, the weaker the functional connectivity. We did not observe such a correlation in the control group. Inhibition is mainly related to the prefrontal cortex (PFC), and the function of the PFC in children with ADHD varies with age (29). Psychological tasks commonly used to study inhibition include Stroop test, the reversed Stroop test, and the go/no-go test. Recently, some studies using the NIRS technique showed that children with ADHD exhibited lower PFC activation than those in the control group when performing the go/no-go task (28, 30), demonstrating a deficit in PFC function among children with ADHD. And similar findings were reported in some studies where the reversed Stroop test was performed (29). Some studies further identified brain regions with reduced activation as the left dorsolateral prefrontal cortex (DLPFC) (17). Children with ADHD had reduced activation in the left DLPFC during the execution of a working memory task measured by NIRS (30). These studies suggest that the prefrontal lobe is indispensable for examining executive functions such as inhibition. NIRS can detect the functioning of the prefrontal lobe, which shows that it is a very appropriate technique for studying neural mechanisms in children with ADHD.

The core defect of ADHD is inhibition dysfunction (3). Patients with ADHD was found to have more pronounced abnormalities of brain function in tasks requiring inhibition than in other tasks, and the brain function was assessed by fMRI (31). A review demonstrated that fNIRS results were very similar to fMRI results in terms of identifying brain functional impairments in inhibition tasks among ADHD individuals (32). However, there were a limited body of literature regarding the relationship between the brain dysfunction in resting state and cognitive/behavioral impairment in children with ADHD. In particular, data was scarce when using fNIRS, an emerging brain imaging technology. fNIRS is a convenient option in clinical application for identifying brain functional abnormalities in ADHD patients due to the ease of using and carrying as well as the relatively low cost. This study has extended our understanding of the neurophysiological mechanism of inhibition function in ADHD children by showing a substantial relationship between inhibition dysfunction and impaired brain functional connectivity.

### 4.1. Strengths and limitations

The present study is one of the few that applied rs-NIRS to verify the correlation between a lack of functional brain connectivity in the whole brain and impaired inhibition function in children with ADHD. However, several limitations warrant attention. First, fNIRS has less spatial resolution than fMRI and can only detect hemodynamic changes on the brain’s surface (about 2–3 cm below the skull). Second, due to the relatively small sample size, this study did not further explore the influence of sex and disease-specific factors on brain connectivity and inhibition. We recommended further studies to extend the sample and assess the association between functional connectivity and inhibition by adjusting a comprehensive set of covariates and confounders. Third, the cross-sectional design of the present study does not allow, the conclusion of causation to be reliably drawn. Prospective cohort studies are needed to illustrate the causal effects.

## 5. Conclusion

Our study demonstrated that children with ADHD had significantly decreased functional brain connectivity in several specific brain regions under the resting state compared with the control group. A lack of functional connectivity was significantly associated with poor inhibition function among children with ADHD.

## Data availability statement

The original data supporting the conclusion of this article will be made available from the first author, WL, upon reasonable request.

## Ethics statement

The studies involving human participants were reviewed and approved by the Medical Ethics Committee of Hangzhou Seventh People’s Hospital. Written informed consent to participate in this study was provided by the participants’ legal guardian/next of kin.

## Author contributions

WL, RY, and GB involved in the study conceptualization and study design. WL and LC collected the data and carried out the statistical analyses. WL and GB wrote the first draft of the manuscript. RY and GB supervised the project. All authors provided the critical revisions of the manuscript for important intellectual content and contributed to the interpretation of the data and approved the final version of the article.
