# Supplementary figures and images for: Lack of functional brain connectivity was associated with poor inhibition in children with attention-deficit/hyperactivity disorder using near-infrared spectroscopy
Source: Front Psychiatry. 2023 Jul 12;14:1221242. doi: 10.3389/fpsyt.2023.1221242 (PMC10368997; doi:10.3389/fpsyt.2023.1221242)

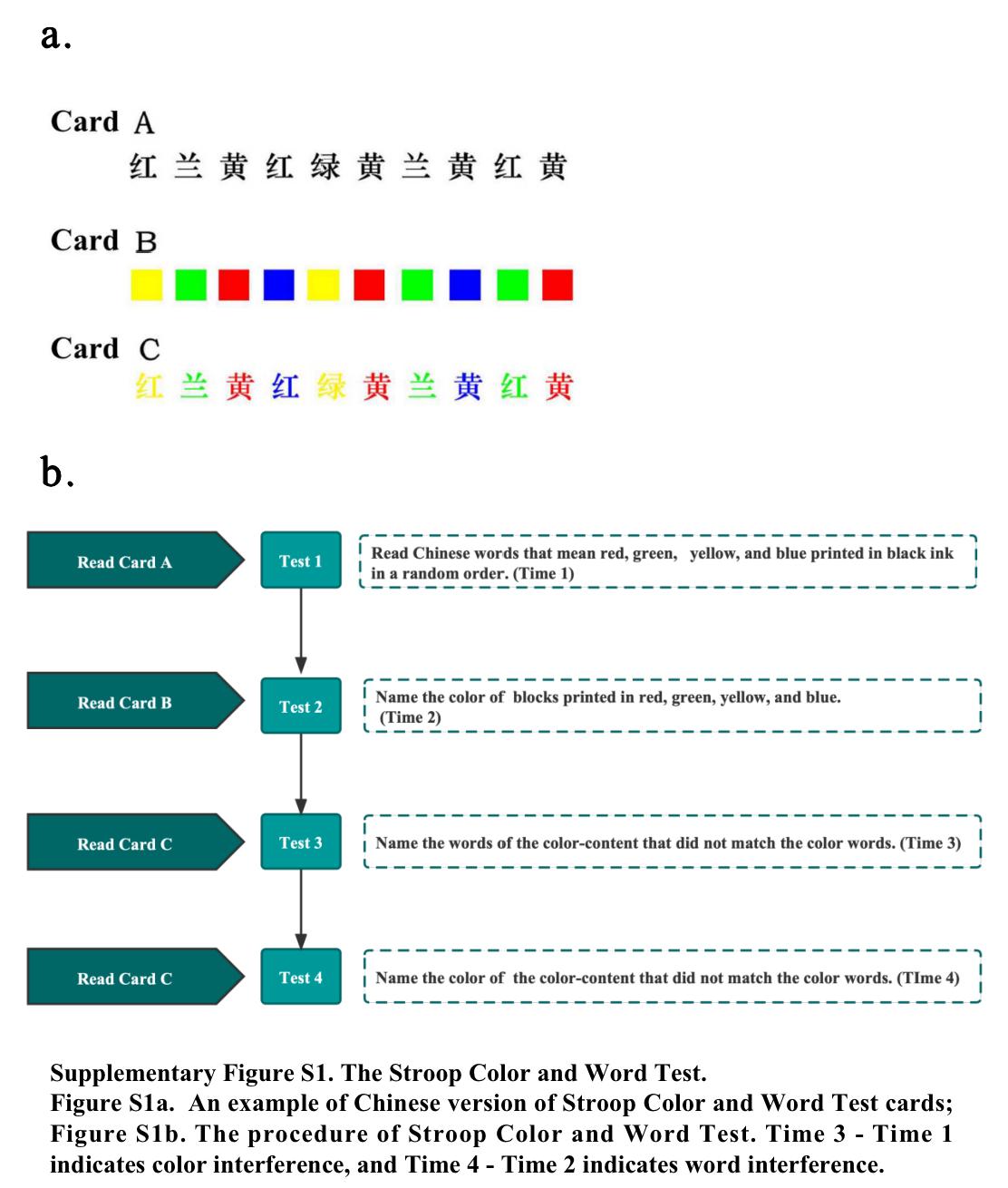

Supplement: Supplementary file 1 [file Image_1.JPEG]
